# Epigallocatechin‐3‐gallate sensitises multidrug‐resistant oral carcinoma xenografts to vincristine sulfate

**DOI:** 10.1002/2211-5463.12905

**Published:** 2020-06-18

**Authors:** Li Chen, Xianwen Guo, Ye Hu, Li Li, Gang Liang, Guo Zhang

**Affiliations:** ^1^ New Drug Research & Development Center First Affiliated Hospital of Guangxi Medical University Nanning China; ^2^ Department of Gastroenterology The People's Hospital of Guangxi Zhuang Autonomous Region Nanning China; ^3^ Pharmacy School of Guangxi Medical University Nanning China; ^4^ Guangxi Medical University Nanning China

**Keywords:** angiogenesis, epigallocatechin‐3‐gallate, multidrug resistance, oral squamous cell carcinoma, tumour growth, vincristine sulfate

## Abstract

Oral squamous cell carcinoma (OSCC) is a very aggressive malignancy, and 50% of patients who receive curative treatment die from the disease or related complications within 5 years. Epigallocatechin‐3‐gallate (EGCG) is the most abundant bioactive ingredient of tea polyphenols in green tea and has anticancer properties. Here, we evaluated the preclinical efficacy of EGCG combined with vincristine sulfate (VCR) on the growth, angiogenic activity and vascular endothelial growth factor (VEGF) expression in xenograft nude mice inoculated with KBV200 cells. Compared with VCR alone, the combined use of EGCG and VCR strongly inhibited tumour growth and angiogenesis (*P* < 0.01). VEGF mRNA and protein levels were lower in the KBV200 xenograft group treated with the combined regime (*P* < 0.01) than those in the VCR alone group. EGCG sensitises multidrug‐resistant OSCC to VCR, and this may occur through the inhibition of angiogenesis via VEGF down‐regulation.

## Graphical abstract

Multidrug resistance is a formidable obstacle to effective chemotherapy of oral squamous cell carcinoma (OSCC). Epigallocatechin‐3‐gallate (EGCG) may be a promising candidate in the quest for overcoming multidrug‐resistant OSCC. EGCG combined with vincristine sulfate (VCR) strongly inhibited tumour growth and angiogenesis *in vivo*. EGCG sensitises multidrug‐resistant OSCC to VCR, and this may occur by inhibiting angiogenesis through VEGF down‐regulation.

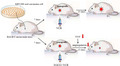

**Abbreviations**
CAM: chick embryo chorioallantoic membrane
DAB: 3,3‐diaminobenzidine
EGCG: epigallocatechin‐3‐gallate
MDR: multidrug resistance
MVD: microvessel density
OSCC: oral squamous cell carcinoma
PCR: polymerase chain reaction
Pgp: P‐glycoprotein
RTV: relative tumour volume
VCR: vincristine sulfate
VEGF: vascular endothelial growth factor

Oral squamous cell carcinoma (OSCC) is a very aggressive malignancy of head and neck cancer and has a dismal outcome, that is, more than 50% of patients who received curative treatment would die from the disease or related complications within 5 years [1, 2, 3, 4, 5]. Effective treatments for OSCC include chemotherapy, surgery and radiotherapy [6]. Although the short‐term efficacy of these treatments is confirmed, the long‐term prognosis of patients remains poor, which is mainly attributed to OSCC recurrence and development of resistance to chemotherapeutic agents.

Multidrug resistance (MDR) is a phenomenon whereby human tumours acquire resistance to multiple anticancer drugs with diverse structures and mechanisms of action; MDR is the main cause of chemotherapy failure and high mortality rate of cancer [7]. One of the important advances in our understanding of MDR is the identification of P‐glycoprotein (Pgp) and other related cell membrane transporters, which can increase the efflux of cytotoxic drugs from cancer cells and thus decrease their intracellular concentrations [6, 7, 8, 9]. Classical MDR is often partly due to the elevated expression of efflux pumps in the cell membrane [10]. The medium of the breast epithelial tumour cell line MCF7, which is known to overexpress MDR1 Pgp, induces angiogenesis correlated with platelet‐activating factor [11]. A new and seemingly counterintuitive preclinical strategy to combat drug resistance in cancer has been developed; in this method, ‘chemotherapeutics act as antiangiogenic agents’, that is the ability of such drugs to damage or kill genetically stable host endothelial cells of a tumour's newly formed neovasculature [12, 13]. The administration of chemotherapeutic drugs in this manner is called ‘antiangiogenic’ or ‘metronomic’ chemotherapy [13].

Epigallocatechin‐3‐gallate (EGCG) is the most abundant bioactive ingredient of tea polyphenols in green tea and exhibits a wide range of pharmacological actions, including anticancer, antiultraviolet radiation and antiangiogenic effects [14, 15, 16, 17]. The anticancer effect of EGCG could be due to its inhibition of tumour angiogenesis by reducing vascular endothelial growth factor (VEGF) expression, resulting in suppressed tumour growth [18, 19, 20]. Pilot studies suggested that EGCG may help reverse the MDR of various human cancers [21, 22, 23]. Yuan *et al*. [24] reported that EGCG sensitises cisplatin‐resistant oral cancer by MDR1 signalling inhibition. However, the effect of EGCG on tumour MDR and the exact mechanisms have not been elucidated, especially the reversible effect of EGCG. Moreover, EGCG enhances the synergistic anticancer effect of other drugs, such as melatonin; as such, combining EGCG with other drugs is a reasonable strategy to treat cancers [25, 26]. However, EGCG has side effects. Mice treated with gradually increasing doses of EGCG exhibited some of the features observed in patients with subacute liver failure, especially ascites [27].

The present study aimed to evaluate the preclinical efficacy of EGCG combined with vincristine sulfate (VCR) in human OSCC. A KBV200 cell‐inoculated xenograft nude mouse model, which is a representative MDR OSCC model, was established. The model was treated with different compounds to examine antitumour activity *in vivo* and investigate the underlying mechanisms. The effects of the combined treatment on tumour growth, angiogenesis and VEGF expression were assessed using daily measurement, immunohistochemistry, ELISA and RT‐PCR analysis.

## Materials and methods

### Materials

The KBV200 human MDR of oral carcinoma cell line was kindly provided by X. Zuoliang (Beijing University, China). BALB/C‐nu/nu nude mice (4–5 weeks old) weighing 13–17 g were obtained from SLRC Laboratory Animal Co., Ltd (Shanghai, China). The mice were maintained under pathogen‐free conditions in facilities approved by the Animal Centre of Guangxi Medical University. EGCG was extracted and separated according to the method of the patent (No: ZL91109820.8). VCR was purchased from Shanghai Hualian Pharmaceutical Company (Shanghai, China). RPMI‐1640 medium and FBS were acquired from Gibco‐BRL Life Technologies, Inc. (Gaithersburg, MD, USA). Anti‐VEGF and anti‐CD34 antibodies and Ultra‐Sensitive™ SP Plus Kit were bought from Fuzhou Maixin Company (Fujian, China). Human VEGF ELISA kit was supplied by Jingmei Biotechnology (Beijing) Co., Ltd (Shenzhen, China). Rapid RNA extraction and purification kit was purchased from Beijing Huashun Biotechnology Company (Beijing, China). First‐strand cDNA synthesis kit was obtained from MBI, Inc. (Salt Lake City, UT, USA). PCR amplification primers were supplied by Shanghai Sangon Biological Engineering Technology and Service Co., Ltd (Shanghai, China). Fertilised white leghorn chicken eggs were provided by Liang Feng Agricultural and Animal Co., Ltd (Nanning, China).

### Chick embryo chorioallantoic membrane assay

Chick embryo chorioallantoic membrane (CAM) assay was performed as described with slight modification [28]. The eggs were soaked in 1% solution of bromo‐geramine for 5–10 min. The fertilised chick eggs were placed on a tray with 45° angle and incubated for 5 days at (37.6 ± 0.2) °C and 60% relative humidity. After incubation, a small window (15 mm × 15 mm) on the air chamber side of the eggshell was opened with a dental saw. Shells were removed carefully with sterile forceps. Subsequently, 80, 160 and 320 μg of EGCG (80, 160 and 320 mg·L^−1^, 1 mL each) and PBS were injected directly onto the CAM. The eggs were sealed with sterile medical tape and incubated for 48 h. The CAM was then fixed with a mixture of methanol and acetone (1 : 1) for 15 min and photographed using a camera. The extent of angiogenesis was determined by counting the number of blood vessels over 5 mm circumambient CAM.

### Cell culture

KBV200 cells were cultured at 37 °C [5% (v/v) CO_2_] *in vitro* by using RPMI‐1640 supplemented with 10% (v/v) FBS, 100 U·mL^−1^ penicillin G and 100 µg·mL^−1^ streptomycin. The culture medium was added with 200 nmol·L^−1^ VCR to maintain VCR resistance. For subculturing, the cells were dissociated using 0.25% trypsin with 0.02% EDTA.

### *In vivo* animal model studies

KBV200 cells were cultured and isolated by typsinisation. Cell number was counted using a haemacytometre. The cells were resuspended in PBS, and 5 × 10^7^ cells per 0.2 mL of PBS were injected into the right axilla of each nude mouse. When the average tumour size reached 125 ± 56 mm^3^ in 8 days, the mice were randomly divided into six groups (*n* = 6 in each group, three males and three females) as follows: control (PBS only), VCR (0.46 mg·kg^−1^, q4d, intraperitoneal injection), EGCG (20 mg·kg^−1^, qd, intraperitoneal injection), VCR combined with low‐dose EGCG (VE low group; 10 mg·kg^−1^, qd, intraperitoneal injection), VCR combined with medium‐dose EGCG (VE mid group; 20 mg·kg^−1^, qd, intraperitoneal injection) and VCR combined with high‐dose EGCG (VE high group; 40 mg·kg^−1^, qd, intraperitoneal injection). Drug doses were determined according to the preliminary experiment in the absence of cytotoxic effect. Tumour dimensions were measured with calipres every 2 days. Mean tumour volume was calculated using the following formula: *V* (cm^3^) = width^2^ × length × 0.52. The mice were sacrificed on day 13 after drug administration. The tumours were resected to measure the weight and calculate tumour inhibition (%) by using the formula (mean weight_control_ − mean weight_drug administration_) × 100%/mean weight_control_. Relative tumour volume (RTV) was determined using *V*_sacrificed_/*V*_before drug administration_, and relative tumour proliferation ratio (%) was measured using RTV_drug administration_/RTV_control._

### Immunohistochemistry and staining of fixed tumour sections

After the mice were sacrificed, tumours were fixed immediately in 10% buffered formalin phosphate and embedded in paraffin. The sections were stained with SP kit in accordance with the manufacturer's instructions. The paraffin sections were routinely dewaxed with xylene and hydrated with gradient alcohol. EDTA buffer antigen retrieval was used for VEGF antigens, and CD34 antigens were citrate buffer retrieved. Primary antibodies (1 : 50) were applied at 4 °C overnight. The sections were washed with PBS and incubated with secondary antibodies at room temperature. The sections were coloured with 3,3‐diaminobenzidine and stained with haematoxylin. The negative control was prepared with the same steps, except that the primary antibody was used instead of PBS.

Any cytoplasmic yellow brown‐stained tumour or endothelial cells were considered as VEGF‐positive cells. Positive rate was evaluated by calculating the percentage of positive cells with a minimum of 500 cells.

Angiogenic activity was assessed by microvessel density (MVD) analysis using immunohistochemistry with antibodies to endothelial marker CD34 and determined according to the method of Foote *et al*. [29]. In brief, CD34‐immunostained sections were firstly observed at low magnification (×100) to identify areas with the highest neovascularisation. Within the areas, the stained microvessels were counted at high magnification (×400). The average count of vessels in five random areas was considered the value of MVD.

### ELISA determination of EGCG

Mouse blood was obtained and centrifuged at   1500 ***g*** for 10 min. Serum was collected and frozen at −80 °C. VEGF concentration was measured using ELISA kits in accordance with the manufacturer's instructions.

### Semiquantitative RT‐PCR analysis of EGCG mRNA expression

The tumour tissue was collected, lysed and processed for total RNA isolation at 4 °C by using an RNA extraction and purification kit. Total RNA concentration in each sample was determined using a spectrophotometer. The integrity of the extracted RNA was confirmed by electrophoresis under denaturing conditions. Total RNA (1 µg) was reverse‐transcribed to cDNA by using the first cDNA synthesis, and primers were chemically synthesised with a DNA synthesiser. The primers used for VEGF and β‐actin gene amplification are shown in Table 1. The PCR products were visualised on 1.5% agarose gel, and pictures were taken with a Bio‐Rad Gel Doc 2000 gel imaging analyser (Bio‐Rad, Hercules, CA, USA). Relative gene expression level was calculated by normalisation to β‐actin by using quantity one analytic software (IBM Corp., Armonk, NY, USA).

**Table 1:** RT‐PCR primer sequences and amplification condition of gene.

Gene	Direction	Sequence (5′–3′)	Product (bp)
VEGF	Sense	CGAAACCATGAACTTTCTGCTGTC	452
	Antisense	TCACCGCCTCGGCTTGTCACAT	584
β‐actin	Sense	ATCTTCAAACCTCCATGATG	120
	Antisense	ACCCCCACTGAAAAAGATGA	120

### Statistical analysis

All statistical analyses were performed using spss 17.0 software package (IBM Corp.). Data are expressed as mean ± SD. Differences between groups were examined using ANOVA for repeated measures, followed by Duncan's multiple range test. *P* values < 0.05 were considered statistically significant.

## Results

### Effect of EGCG on angiogenesis in CAM

Chick embryo chorioallantoic membrane was used to analyse the effect of EGCG on angiogenesis. Newborn vessels in the PBS group were rich, clear and have intact branching structure. Despite no significant morphological difference in large vessels between EGCG and PBS groups, the results indicated that EGCG exhibited substantial growth inhibitory effect on medium and small vessels, especially on the latter (*P* < 0.01, Table 2).

**Table 2:** Effect of EGCG on angiogenesis in CAM. Data expressed as mean ± SD.

Group	Large vessel	Medium vessel	Small vessel
PBS	2.20 ± 0.79	5.70 ± 1.34	21.60 ± 3.06
EGCG (80 mg·L^−1^)	2.10 ± 0.57	5.00 ± 0.94	18.00 ± 1.63^**^
EGCG (160 mg·L^−1^)	2.10 ± 0.74	4.90 ± 0.74	14.30 ± 2.00^**^
EGCG (320 mg·L^−1^)	2.00 ± 0.67	4.60 ± 1.07^*^	11.60 ± 1.96^**^

* *P* < 0.05 and **P < 0.01 *vs*. PBS group (one‐way analysis of variance, *n* = 10 in each group).

### Effect of EGCG on tumour growth in KBV200 xenograft model

**Fig. 1:**
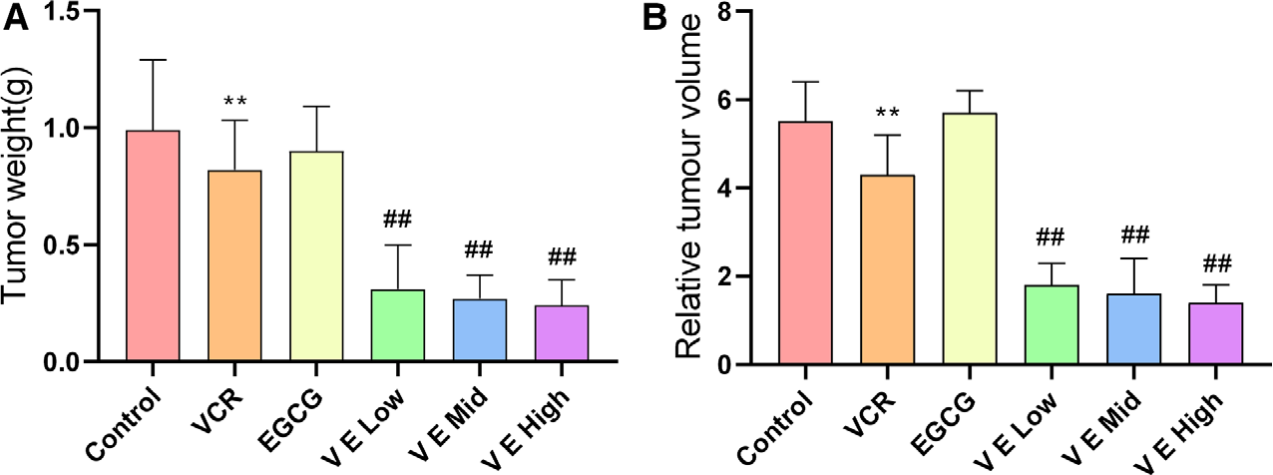
Epigallocatechin‐3‐gallate and VCR‐combined treatment considerably inhibited tumour growth in KBV200 xenograft. (A) Tumour weight. (B) Relative tumour volume. A KBV200 cell‐inoculated nude mice xenograft, which is a representative MDR OSCC model, was established and then randomly divided into six groups treated with different compounds as follows: control (PBS only), VCR alone (0.46 mg·kg^−1^), EGCG alone (20 mg·kg^−1^), VCR combined with low‐dose EGCG (VE low group; 10 mg·kg^−1^), VCR combined with medium‐dose EGCG (VE mid group; 20 mg·kg^−1^) and VCR combined with high‐dose EGCG (VE high group; 40 mg·kg^−1^). Compared with the VCR group, the combination of EGCG and VCR could considerably inhibit tumour growth in a dose‐dependent manner, while EGCG alone could not. Data expressed as mean ± SD.**P < 0.01 *vs*. control group, *^##^P* < 0.01 vs. VCR group (one‐way analysis of variance, *n* = 6).

Our group and other researchers reported that EGCG can inhibit tumour growth in a variety of human cancers [30, 31, 32]. Here, we observed a similar effect on the multidrug‐resistant KBV200 xenograft model. Figure 1 shows that EGCG had no evident effect on tumour weight and volume compared with the control group, thereby revealing that EGCG alone could not inhibit KBV200 growth. VCR could suppress tumour growth, and this effect was greatly enhanced by combining VCR with EGCG. Hence, the combination of EGCG and VCR could significantly inhibit tumour growth. By contrast, tumour growth was inhibited in the EGCG combined with the VCR group. The RTV decreased from 1.8 ± 0.5 to 1.4 ± 0.4, while the concentration of EGCG increased. Thus, EGCG combined with VCR inhibited tumour growth in a dose‐dependent manner (Table 3).

**Table 3:** Effect of EGCG on VCR anticancer activity in KBV200 xenograft nude mice. Data expressed as mean ± SD.

Group	Tumour weight (g)	IR (%)	RTV	T/C (%)
Control	0.99 ± 0.30	–	5.5 ± 0.9	100
VCR	0.82 ± 0.21^**^	17.6	4.3 ± 0.9^**^	77.7
EGCG	0.90 ± 0.19	9.5	5.7 ± 0.5	103.5
V E low	0.31 ± 0.19***^##^***	69.1	1.8 ± 0.5***^##^***	33.3
V E mid	0.27 ± 0.10***^##^***	72.8	1.6 ± 0.8***^##^***	29.4
V E high	0.24 ± 0.11***^##^***	76.1	1.4 ± 0.4***^##^***	25.2

* *P* < 0.05 and **P < 0.01 *vs*. control group, *^#^P* < 0.05 and *^##^P* < 0.01 vs. VCR group (one‐way analysis of variance, *n* = 6).

All animals survived after the experimental model procedures. The evolution of body weights of the six study groups is shown in Table 4. Except for the VCR group, there was a gradual weight gain in other five groups over 13 days. The higher weight gain was observed in the control group mice, with no significant difference between the EGCG groups (*P* > 0.05). In addition, VCR possessed toxicity to mice, showing a significant loss of body weight (*P* < 0.01). However, EGCG can reduce the toxicity of VCR, showing a significant gain of body weight (*P* < 0.01). These results suggest that EGCG could sensitise multidrug‐resistant OSCC to VCR *in vivo*.

**Table 4:** Effect of EGCG on VCR toxicity in KBV200 xenograft nude mice. Data expressed as mean ± SD.

Group	Mice survived (*n*) initial/end	Body weight (g) initial/end
Control	6/6	16.5 ± 0.49/18.5 ± 0.19
VCR	6/6	16.58 ± 0.7/15.8 ± 0.51^**^
EGCG	6/6	16.57 ± 0.74/18.45 ± 0.4***^##^***
V E low	6/6	16.48 ± 0.55/17.68 ± 0.38^**##^
V E mid	6/6	16.47 ± 0.56/17.83 ± 0.33^**##^
V E high	6/6	16.45 ± 0.91/18.03 ± 0.47^*##^

* *P* < 0.05 and **P < 0.01 *vs*. control group, *^#^P* < 0.05 and *^##^P* < 0.01 vs. VCR group (one‐way analysis of variance).

### Effect of drug on MVD in KBV200 xenograft nude mice

**Fig. 2:**
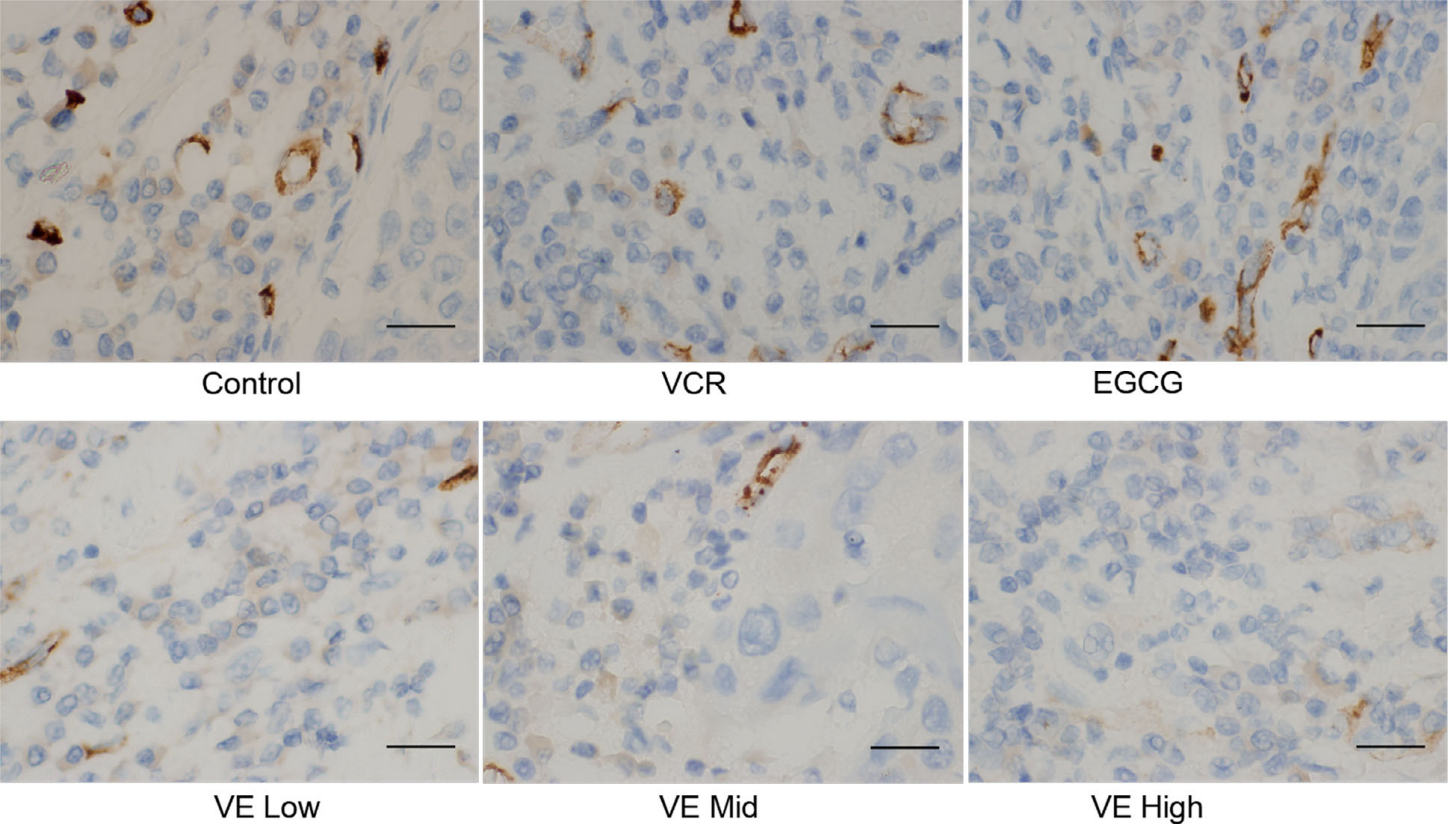
Combination of EGCG and VCR exhibited stronger inhibitory effect on tumour angiogenesis. Angiogenic activity was assessed by MVD analysis using immunohistochemistry with antibodies to the endothelial marker CD34 (*n* = 6 in each group, magnification of ×400). In brief, the CD34‐immunostained sections were firstly observed at low magnification (×100) to identify the areas of highest neovascularisation. Within the areas, the stained microvessels were counted at high magnification (×400), and the average vessel count in five random areas was considered as the MVD value. The MVD in the control group was significantly higher than that in the VCR group (*P* < 0.01). However, the MVD in the combined treatment was much lower than that in the VCR group (*P* < 0.05), indicating that EGCG combined with VCR exhibited stronger inhibitory effect on tumour angiogenesis. Scale bar: 20 µm.

As shown in Fig. 2, the variations in microvascular morphology and size were different. The figure also displays endothelial cell clusters or single endothelial cell with a ring‐shaped or cable structure. Dense microvascular morphology was mostly distributed within the front area infiltrated by tumour cells. As shown in Table 5, the MVD in the control group was 22.58 ± 3.83, which was significantly higher than that in the VCR group (*P* < 0.01). However, the MVD in the combined treatment group was lower than that in the VCR group (*P* < 0.05).

**Table 5:** Effect of drug on MVD in KBV200 xenograft nude mice. Data expressed as mean ± SD.

Group	MVD (number/×400)
Control	22.58 ± 3.83
VCR	18.67 ± 3.19^**^
EGCG	21.53 ± 3.73
V E low	15.83 ± 2.14^#*^
V E mid	12.07 ± 1.88***^##^***
V E high	9.25 ± 1.91***^##^***

* *P* < 0.05 and **P < 0.01 *vs*. control group, *^#^P* < 0.05 and *^##^P* < 0.01 vs. VCR group (one‐way analysis of variance, *n* = 6).

### Effect of EGCG on VEGF expression in KBV200 xenograft model

**Fig. 3:**
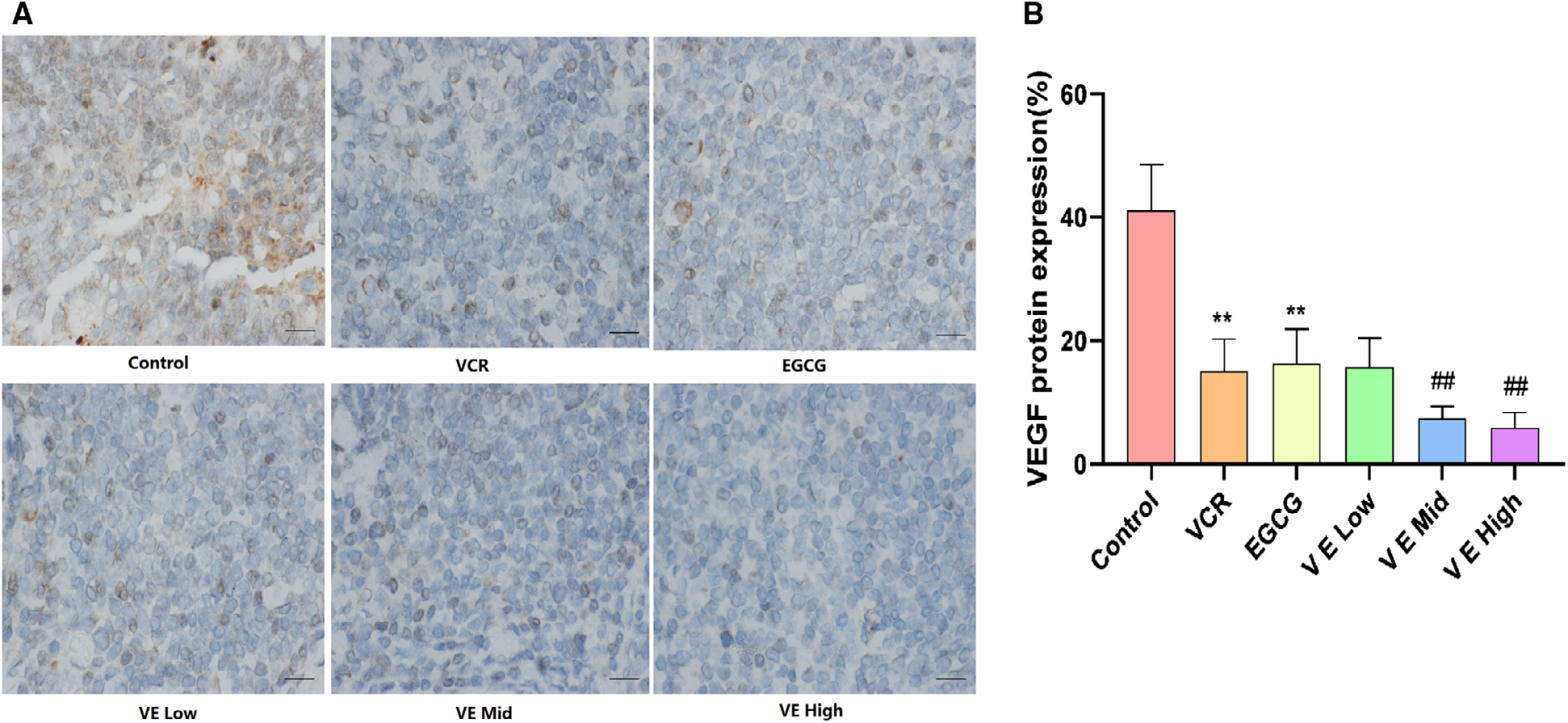
Effect of EGCG on VEGF expression in KBV200 xenograft model. (A) Representative immunohistochemical staining image of tumour tissue (magnification of ×400). Scale bar: 20 µm. (B) Summarised data. The VEGF protein expression in tumour tissues, which displayed brown diffuse cytoplasmic distribution in cells, was more distributed around the edge of the active growth cells than at the centre. The VEGF expression was decreased by EGCG and VCR, and a synergistic effect existed when the two were combined with each other. The VEGF expression in low‐dose EGCG combined with VCR did not significantly differ from that in the VCR group. However, the VEGF expression in mid‐ and high‐dose EGCG combined with VCR exhibited a marked reduction compared with that in the VCR group. Data expressed as mean ± SD.**P < 0.01 *vs*. control group, *^##^P* < 0.01 vs. VCR group (one‐way analysis of variance, *n* = 6).

As shown in Fig. 3, the VEGF protein expression in tumour tissues, which displayed brown diffuse cytoplasmic distribution in cells, was distributed more around the edge of the active growth cells than that at the centre. The VEGF expression levels in different treatment groups were as follows: control, 41.17%; VCR, 15.12%; EGCG, 16.34%; and VE mid, 7.41%. ANOVA showed that the VEGF expression was decreased by EGCG and VCR, and a synergistic effect existed when the two were combined (*P* < 0.01). The VEGF expression in the VE low group did not significantly differ from that in the VCR group. However, the VEGF expression levels in the mid and high VE groups were reduced compared with that in the VCR group (*P* < 0.01).

### Effect of EGCG on VEGF serum levels in KBV200 xenograft model

**Fig. 4:**
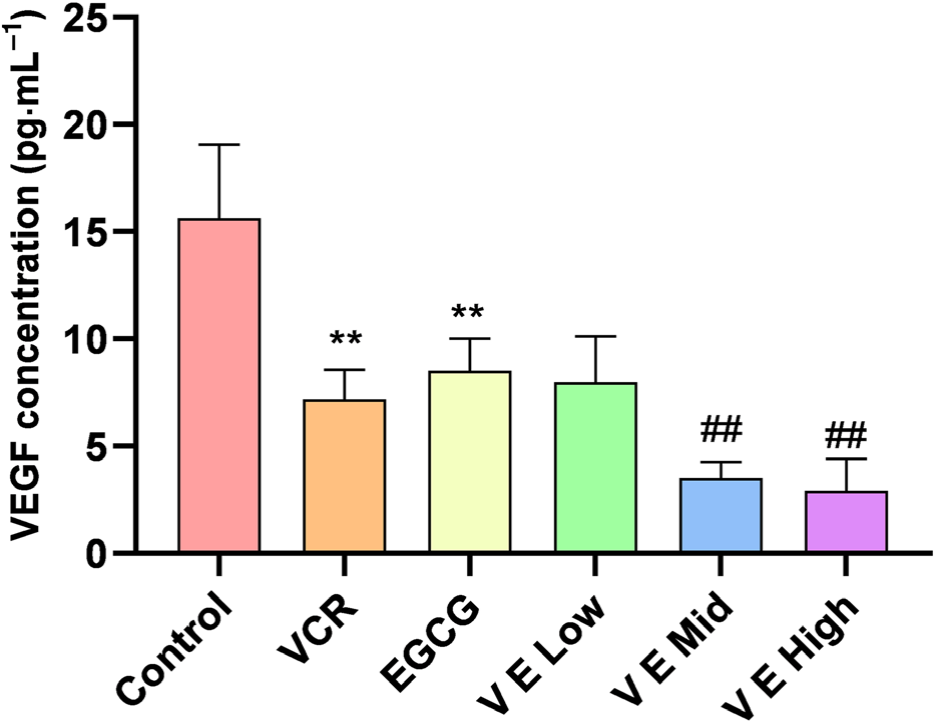
Effect of EGCG on VEGF serum levels in KBV200 xenograft model. The serum levels of VEGF were measured using ELISA. The VEGF concentration of either the VCR or EGCG group was significantly decreased compared with that of the control group. The VEGF level in the VE mid and high groups was evidently further decreased compared with that in the VCR group (*P* < 0.01). Data expressed as mean ± SD. **P < 0.01 *vs*. control group, *^##^P* < 0.01 vs. VCR group (one‐way analysis of variance, *n* = 6).

The serum levels of VEGF were measured using ELISA. As shown in Fig. 4, the VEGF concentration in the VCR and EGCG groups significantly decreased compared with that in the control group (*P* < 0.01). The serum concentration levels of VEGF were 3.52 ± 0.75 and 2.93 ± 1.49 pg·mL^−1^ in the mid and high VE groups, respectively. These values were obviously decreased (*P* < 0.01) compared with those in the VCR group.

### Effect of EGCG on VEGF mRNA expression in KBV200 xenograft model

**Fig. 5:**
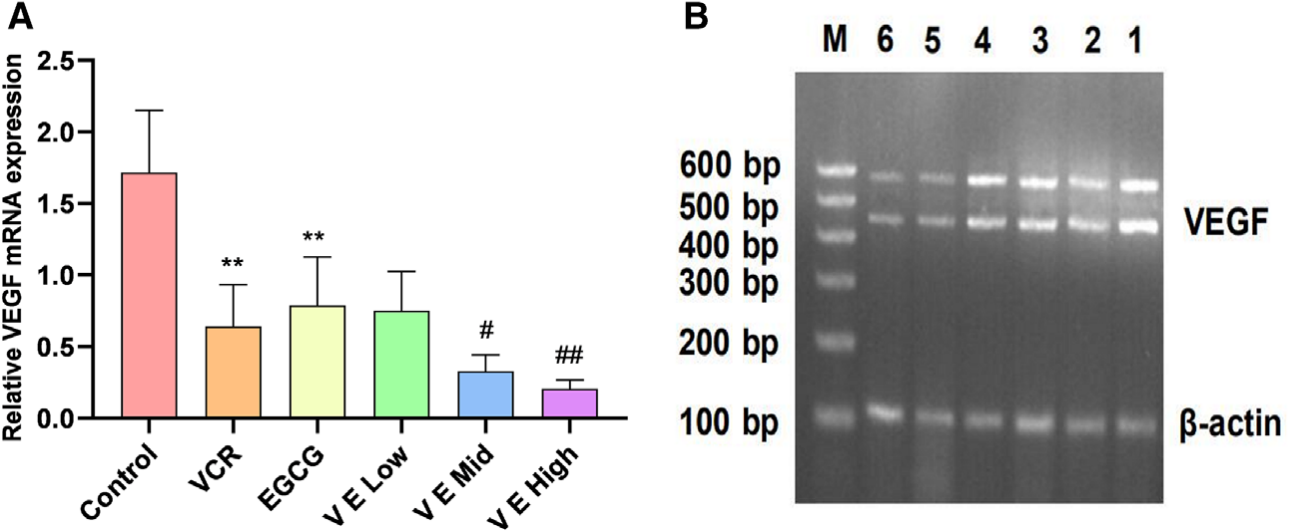
Effect of EGCG on VEGF mRNA expression in KBV200 xenograft model. (A) Summarised data showing the effect of drug on VEGF mRNA expression. (B) Representative gel image of PCR production: lane 1, control; lane 2, VCR; lane 3, EGCG; lane 4, VE low; lane 5, VE mid; lane 6, VE high; and M, maker. The mRNA levels of VEGF in different groups were measured via RT‐PCR. The results indicated that EGCG had similar effects on VEGF mRNA and serum levels. Combining EGCG with VCR could significantly inhibit VEGF mRNA expression compared with VCR alone, except in the low EGCG‐combined group (*P* < 0.05). Data expressed as mean ± SD. **P < 0.01 *vs*. control group, *^#^P* < 0.05 and *^##^P* < 0.01 vs. VCR group (one‐way analysis of variance, *n* = 6).

The mRNA of VEGF in the tumour tissues of different treatment groups was measured by RT‐PCR to further examine the involvement of EGCG in the regulation of VEGF at the mRNA level. EGCG demonstrated similar effects on VEGF mRNA and serum levels. As shown in Fig. 5, the combination of EGCG and VCR significantly inhibited VEGF mRNA expression compared with VCR alone, except in the low EGCG group (*P* < 0.05).

## Discussion

Multidrug resistance is a critical cellular defence mechanism that protects tumour cells from chemotherapy agents; this phenomenon is the major cause of treatment failure in patients with cancer [33]. Drug efflux transporters have emerged as a research hotspot. For example, Pgp is one of the protein families located in the plasma membrane and is encoded by the MDR1 gene [33, 34]. Other molecules that are involved in drug resistance include MDR‐associated proteins [35, 36], glutathione S‐transferase‐p [37] and DNA topoisomerase II [38]. However, these molecules cannot fully explain MDR in OSCC. MDR is an extremely complex and multifactorial process [7]. Neovascularisation plays an important role in MDR. MDR phenotypes are closely associated with the phenotype of tumour angiogenesis in human hepatoma cell lines [39]. VEGF is the most crucial factor during the formation of tumour blood vessels [40]. Klement *et al*. [41] found that anti‐VEGFR‐2 antibodies combined with a small dose of chemotherapy drugs could reverse the MDR of high Pgp‐expressing breast cancer cells. VEGFR‐2 tyrosine kinase inhibitor YM‐231146 could completely suppress the growth of taxol‐resistant tumour at the dose of 100 mg·kg^−1^; this finding indicates the huge effect of VEGF on MDR [42].

Researchers have been searching for resistance reversal agents that have high efficiency, low toxicity and a wide range of targets. Although compounds, such as verapamil and tamoxifen, demonstrate resistance reversal activity in clinical applications, they still have limitations owing to the narrow spectrum of targets and considerable side effects [43, 44, 45]. Chinese medicine has its superiority. Most Chinese medicines possess antitumour effect; moreover, their effect can be enhanced and their toxicity can be attenuated when combined with chemotherapy drugs. These medicines are multitargeting and have limited adverse reactions. Many extracts from Chinese herbals, monomer compositions or compounds, such as tea polyphenols, tetrandrine, neferine and *Brucea javanica* oil emulsion, could reverse the MDR of leukaemia, breast cancer, liver cancer, gastric cancer and oral epithelial carcinoma [46, 47]. Therefore, Chinese medicine that can reverse MDR has become the focus of antitumour drug research.

Epigallocatechin‐3‐gallate, the main component of tea polyphenols, exhibits powerful biological effects, such as antimutation, antitumour formation, MDR reversal and anti‐angiogenesis [48, 49]. The exact mechanisms of the multiple pharmacological effects of EGCG involve a number of key enzymes and information transmission [50]. The anti‐angiogenesis effect of EGCG is fundamental to its antitumour formation. Experiments verified that EGCG inhibits CAM vascular growth at the dose of 80–320 mg·L^−1^. EGCG can also inhibit the secretion of VEGF in tumour cells, resulting in suppressed tumour angiogenesis and restrained tumour growth [18, 51].

Sustained angiogenesis is a critical factor during the growth and metastasis of solid tumours. After a solid tumour is formed, nutrients for tumour cells are mainly supplied by diffusion in the stage called avascular early stage of invasion. The diameter of tumour nodule is generally less than 2–3 mm during this period. Once neovascularisation occurred inside the tumour, the tumour growth is accelerated immediately [52]. The tumour then becomes prone to invasion and metastasis. Angiogenesis is similar in normal tissues and tumours, but the ratio of immature vessels is higher in the latter due to its particular microenvironmental conditions, including incomplete basement membrane, few intercellular connections, irregular lumen, evident distortion and sinus‐shaped dilation of blood vessels, high degree of atypia, fast growth and abundant vas capillary [53]. The activation of tumour angiogenesis does not mean improved blood perfusion. Meanwhile, immature and nonfunctional vessels, unbalanced distribution of blood vessels and rapid proliferation of tumour cells lead to a hypoxic state [53]. The decline in perfusion leads to difficulty in achieving high concentration in tumour parenchyma; thus, the treatment effect is greatly reduced. At present, MVD is considered the gold standard for the evaluation of tumour angiogenesis. In the present study, we determined MVD by using specific CD34 antibody to mark endothelial cells in tumour. The MVD was considerably reduced to 15.83 ± 2.14, 12.07 ± 1.88 and 9.25 ± 1.91 in the combined EGCG and VCR groups in a dose‐dependent manner compared with that in the VCR group alone (MVD: 18.67 ± 3.19). Given the difference in xenograft growth and tumour apoptosis, EGCG was proven to exert a sensitisation effect on VCR by inhibiting KBV200 growth *in vivo* possibly by partly diminishing blood vessels. This phenomenon blocks nutritional and paracrinal effects on tumour cells and thus induces apoptosis.

However, the effect of EGCG on tumour growth differed from that on VEGF expression. We speculated that the difference could be due to two reasons. Firstly, VEGF detection was performed using immunohistochemistry, which is not sensitive to VEGF. Secondly, the effects of EGCG on tumour growth and VEGF possibly vary, that is, the inhibition of EGCG on tumour growth might be through an alternative pathway in addition to VEGF inhibition. However, the exact reason remains unknown and must be validated in future studies.

Epigallocatechin‐3‐gallate exhibits potential as a new target for cancer therapy. Inhibiting tumour angiogenesis could be an effective therapeutic option to treat refractory chemotherapeutic drug‐resistant tumours.

## Conflict of interest

The authors declare no conflict of interest.

## Author contributions

LC, XG and YH wrote the manuscript. LC, GL and GZ made substantial contributions to the conception, design and intellectual content of the studies. LC, XG, YH, LL and GZ made key contributions to the analysis and interpretation of data. All authors read and approved the final manuscript and agreed to be accountable for all aspects of the research in ensuring that the accuracy or integrity of any part of the work is appropriately investigated and resolved.

## Data Availability

The datasets used are available from the corresponding author upon reasonable request.

## References

[1] Afzali P and Ward BB (2019) Management of the neck in oral squamous cell carcinoma: background, classification, and current philosophy. Oral Maxillofac Surg Clin North Am 31, 69–84. 30449527 10.1016/j.coms.2018.09.004

[2] Argiris A , Karamouzis MV , Raben D and Ferris RL (2008) Head and neck cancer. Lancet 371, 1695–1709. 18486742 10.1016/S0140-6736(08)60728-X PMC7720415

[3] Ferlay J , Soerjomataram I , Dikshit R , Eser S , Mathers C , Rebelo M , Parkin DM , Forman D and Bray F (2015) Cancer incidence and mortality worldwide: sources, methods and major patterns in GLOBOCAN 2012. Int J Cancer 136, E359–E386. 25220842 10.1002/ijc.29210

[4] Li J , Feng X , Sun C , Zeng X , Xie L , Xu H , Li T , Wang R , Xu X , Zhou X *et al* (2015) Associations between proteasomal activator PA28gamma and outcome of oral squamous cell carcinoma: evidence from cohort studies and functional analyses. EBioMedicine 2, 851–858. 26425691 10.1016/j.ebiom.2015.07.004 PMC4563126

[5] Panzarella V , Pizzo G , Calvino F , Compilato D , Colella G and Campisi G (2014) Diagnostic delay in oral squamous cell carcinoma: the role of cognitive and psychological variables. Int J Oral Sci 6, 39–45. 24287962 10.1038/ijos.2013.88 PMC3967306

[6] Balaji SM (2015) Oral squamous cell carcinoma: advances in management. Indian J Dent Res 26, 559. 26888230 10.4103/0970-9290.176889

[7] Baguley BC (2010) Multiple drug resistance mechanisms in cancer. Mol Biotechnol 46, 308–316. 20717753 10.1007/s12033-010-9321-2

[8] Li D , Zhou L , Huang J and Xiao X (2016) Effect of multidrug resistance 1/P‐glycoprotein on the hypoxia‐induced multidrug resistance of human laryngeal cancer cells. Oncol Lett 12, 1569–1574. 27446473 10.3892/ol.2016.4749 PMC4950858

[9] Xue X , Chen F , Liu A , Sun D , Wu J , Kong F , Luan Y , Qu X and Wang R (2016) Reversal of the multidrug resistance of human ileocecal adenocarcinoma cells by acetyl‐11‐keto‐beta‐boswellic acid via downregulation of P‐glycoprotein signals. Biosci Trends 10, 392–399. 27545217 10.5582/bst.2016.01115

[10] Othman RT , Kimishi I , Bradshaw TD , Storer LC , Korshunov A , Pfister SM , Grundy RG , Kerr ID and Coyle B (2014) Overcoming multiple drug resistance mechanisms in medulloblastoma. Acta Neuropathol Commun 2, 57. 24887326 10.1186/2051-5960-2-57 PMC4229867

[11] Mechetner E , Kyshtoobayeva A , Zonis S , Kim H , Stroup R , Garcia R , Parker RJ and Fruehauf JP (1998) Levels of multidrug resistance (MDR1) P‐glycoprotein expression by human breast cancer correlate with in vitro resistance to taxol and doxorubicin. Clin Cancer Res 4, 389–398. 9516927

[12] Miller KD , Sweeney CJ and Sledge GW Jr (2001) Redefining the target: chemotherapeutics as antiangiogenics. J Clin Oncol 19, 1195–1206. 11181686 10.1200/JCO.2001.19.4.1195

[13] Browder T , Butterfield CE , Kraling BM , Shi B , Marshall B , O'Reilly MS and Folkman J (2000) Antiangiogenic scheduling of chemotherapy improves efficacy against experimental drug‐resistant cancer. Cancer Res 60, 1878–1886. 10766175

[14] Zhu W , Jia L , Chen G , Zhao H , Sun X , Meng X , Zhao X , Xing L , Yu J and Zheng M (2016) Epigallocatechin‐3‐gallate ameliorates radiation‐induced acute skin damage in breast cancer patients undergoing adjuvant radiotherapy. Oncotarget 7, 48607–48613. 27224910 10.18632/oncotarget.9495 PMC5217042

[15] Granja A , Pinheiro M and Reis S (2016) Epigallocatechin gallate nanodelivery systems for cancer therapy. Nutrients 8, 307. 10.3390/nu8050307 PMC4882719 27213442

[16] Li MJ , Yin YC , Wang J and Jiang YF (2014) Green tea compounds in breast cancer prevention and treatment. World J Clin Oncol 5, 520–528. 25114865 10.5306/wjco.v5.i3.520 PMC4127621

[17] Lee PMY , Ng CF , Liu ZM , Ho WM , Lee MK , Wang F , Kan HD , He YH , Ng SSM , Wong SYS *et al* (2017) Reduced prostate cancer risk with green tea and epigallocatechin 3‐gallate intake among Hong Kong Chinese men. Prostate Cancer Prostatic Dis 20, 318–322. 28417981 10.1038/pcan.2017.18

[18] Luo HQ , Xu M , Zhong WT , Cui ZY , Liu FM , Zhou KY and Li XY (2014) EGCG decreases the expression of HIF‐1alpha and VEGF and cell growth in MCF‐7 breast cancer cells. J BUON 19, 435–439. 24965403

[19] Fu JD , Yao JJ , Wang H , Cui WG , Leng J , Ding LY and Fan KY (2019) Effects of EGCG on proliferation and apoptosis of gastric cancer SGC7901 cells via down‐regulation of HIF‐1alpha and VEGF under a hypoxic state. Eur Rev Med Pharmacol Sci 23, 155–161. 30657557 10.26355/eurrev_201901_16759

[20] Nabekura T (2010) Overcoming multidrug resistance in human cancer cells by natural compounds. Toxins (Basel) 2, 1207–1224. 22069634 10.3390/toxins2061207 PMC3153241

[21] Wang P , Henning SM , Heber D and Vadgama JV (2015) Sensitization to docetaxel in prostate cancer cells by green tea and quercetin. J Nutr Biochem 26, 408–415. 25655047 10.1016/j.jnutbio.2014.11.017 PMC4375039

[22] Wang S , Chen R , Zhong Z , Shi Z , Chen M and Wang Y (2014) Epigallocatechin‐3‐gallate potentiates the effect of curcumin in inducing growth inhibition and apoptosis of resistant breast cancer cells. Am J Chin Med 42, 1279–1300. 25242081 10.1142/S0192415X14500803

[23] Xiang GL , Zhu XH , Lin CZ , Wang LJ , Sun Y , Cao YW and Wang FF (2017) 125I seed irradiation induces apoptosis and inhibits angiogenesis by decreasing HIF‐1alpha and VEGF expression in lung carcinoma xenografts. Oncol Rep 37, 3075–3083. 28339070 10.3892/or.2017.5521

[24] Yuan CH , Horng CT , Lee CF , Chiang NN , Tsai FJ , Lu CC , Chiang JH , Hsu YM , Yang JS and Chen FA (2017) Epigallocatechin gallate sensitizes cisplatin‐resistant oral cancer CAR cell apoptosis and autophagy through stimulating AKT/STAT3 pathway and suppressing multidrug resistance 1 signaling. Environ Toxicol 32, 845–855. 27200496 10.1002/tox.22284

[25] Wang J , Wang X , He Y , Jia L , Yang CS , Reiter RJ and Zhang J (2019) Antioxidant and pro‐oxidant activities of melatonin in the presence of copper and polyphenols in vitro and in vivo. Cells 8, 903. 10.3390/cells8080903 PMC6721667 31443259

[26] Zhang L , He Y , Wu X , Zhao G , Zhang K , Yang CS , Reiter RJ and Zhang J (2019) Melatonin and (‐)‐epigallocatechin‐3‐gallate: partners in fighting cancer. Cells 8, 745. 10.3390/cells8070745 PMC6678710 31331008

[27] Wang X , Yang L , Wang J , Zhang Y , Dong R , Wu X , Yang CS , Zhang Z and Zhang J (2019) A mouse model of subacute liver failure with ascites induced by step‐wise increased doses of (‐)‐epigallocatechin‐3‐gallate. Sci Rep 9, 18102. 31792332 10.1038/s41598-019-54691-0 PMC6888815

[28] Kue CS , Tan KY , Lam ML and Lee HB (2015) Chick embryo chorioallantoic membrane (CAM): an alternative predictive model in acute toxicological studies for anti‐cancer drugs. Exp Anim 64, 129–138. 25736707 10.1538/expanim.14-0059 PMC4427727

[29] Foote RL , Weidner N , Harris J , Hammond E , Lewis JE , Vuong T , Ang KK and Fu KK (2005) Evaluation of tumor angiogenesis measured with microvessel density (MVD) as a prognostic indicator in nasopharyngeal carcinoma: results of RTOG 9505. Int J Radiat Oncol Biol Phys 61, 745–753. 15708253 10.1016/j.ijrobp.2004.07.694

[30] Chen L , Ye HL , Zhang G , Yao WM , Chen XZ , Zhang FC and Liang G (2014) Autophagy inhibition contributes to the synergistic interaction between EGCG and doxorubicin to kill the hepatoma Hep3B cells. PLoS One 9, e85771. 24465696 10.1371/journal.pone.0085771 PMC3897495

[31] Kwak TW , Park SB , Kim HJ , Jeong YI and Kang DH (2017) Anticancer activities of epigallocatechin‐3‐gallate against cholangiocarcinoma cells. Onco Targets Ther 10, 137–144. 28053547 10.2147/OTT.S112364 PMC5189709

[32] Zhang J , Lei Z , Huang Z , Zhang X , Zhou Y , Luo Z , Zeng W , Su J , Peng C and Chen X (2016) Epigallocatechin‐3‐gallate(EGCG) suppresses melanoma cell growth and metastasis by targeting TRAF6 activity. Oncotarget 7, 79557–79571. 27791197 10.18632/oncotarget.12836 PMC5346735

[33] Kesharwani SS , Kaur S , Tummala H and Sangamwar AT (2018) Overcoming multiple drug resistance in cancer using polymeric micelles. Expert Opin Drug Deliv 15, 1127–1142. 30324813 10.1080/17425247.2018.1537261

[34] Darby RA , Callaghan R and McMahon RM (2011) P‐glycoprotein inhibition: the past, the present and the future. Curr Drug Metab 12, 722–731. 21434857 10.2174/138920011798357006

[35] Yu P , Du Y , Cheng X , Yu Q , Huang L and Dong R (2014) Expression of multidrug resistance‐associated proteins and their relation to postoperative individualized chemotherapy in gastric cancer. World J Surg Oncol 12, 307. 25304659 10.1186/1477-7819-12-307 PMC4198758

[36] Sidjabat HE , Townsend KM , Lorentzen M , Gobius KS , Fegan N , Chin JJ , Bettelheim KA , Hanson ND , Bensink JC and Trott DJ (2006) Emergence and spread of two distinct clonal groups of multidrug‐resistant *Escherichia coli* in a veterinary teaching hospital in Australia. J Med Microbiol 55, 1125–1134. 16849734 10.1099/jmm.0.46598-0

[37] Tew KD , Manevich Y , Grek C , Xiong Y , Uys J and Townsend DM (2011) The role of glutathione S‐transferase P in signaling pathways and S‐glutathionylation in cancer. Free Radic Biol Med 51, 299–313. 21558000 10.1016/j.freeradbiomed.2011.04.013 PMC3125017

[38] Li H , Xie N , Gleave ME and Dong X (2015) Catalytic inhibitors of DNA topoisomerase II suppress the androgen receptor signaling and prostate cancer progression. Oncotarget 6, 20474–20484. 26009876 10.18632/oncotarget.4105 PMC4653019

[39] Lasagna N , Fantappie O , Solazzo M , Morbidelli L , Marchetti S , Cipriani G , Ziche M and Mazzanti R (2006) Hepatocyte growth factor and inducible nitric oxide synthase are involved in multidrug resistance‐induced angiogenesis in hepatocellular carcinoma cell lines. Cancer Res 66, 2673–2682. 16510587 10.1158/0008-5472.CAN-05-2290

[40] Wang L , Zhou R , Zhao Y , Dong S , Zhang J , Luo Y , Huang N , Shi M , Bin J , Liao Y *et al* (2016) MACC‐1 promotes endothelium‐dependent angiogenesis in gastric cancer by activating TWIST1/VEGF‐A signal pathway. PLoS One 11, e0157137. 27280289 10.1371/journal.pone.0157137 PMC4900635

[41] Klement G , Huang P , Mayer B , Green SK , Man S , Bohlen P , Hicklin D and Kerbel RS (2002) Differences in therapeutic indexes of combination metronomic chemotherapy and an anti‐VEGFR‐2 antibody in multidrug‐resistant human breast cancer xenografts. Clin Cancer Res 8, 221–232. 11801563

[42] Amino N , Ideyama Y , Yamano M , Kuromitsu S , Tajinda K , Samizu K , Matsuhisa A , Shirasuna K , Kudoh M and Shibasaki M (2005) YM‐231146, a novel orally bioavailable inhibitor of vascular endothelial growth factor receptor‐2, is effective against paclitaxel resistant tumors. Biol Pharm Bull 28, 2096–2101. 16272696 10.1248/bpb.28.2096

[43] Qin M , Lee YE , Ray A and Kopelman R (2014) Overcoming cancer multidrug resistance by codelivery of doxorubicin and verapamil with hydrogel nanoparticles. Macromol Biosci 14, 1106–1115. 24771682 10.1002/mabi.201400035

[44] Mao Z , Zhou J , Luan J , Sheng W , Shen X and Dong X (2014) Tamoxifen reduces P‐gp‐mediated multidrug resistance via inhibiting the PI3K/Akt signaling pathway in ER‐negative human gastric cancer cells. Biomed Pharmacother 68, 179–183. 24184201 10.1016/j.biopha.2013.10.003

[45] Kim SS , Seong S and Kim SY (2014) Synergistic effect of ginsenoside Rg3 with verapamil on the modulation of multidrug resistance in human acute myeloid leukemia cells. Oncol Lett 7, 1265–1269. 24944704 10.3892/ol.2014.1826 PMC3961386

[46] Jia L , Li Z , Shen J , Zheng D , Tian X , Guo H and Chang P (2015) Multifunctional mesoporous silica nanoparticles mediated co‐delivery of paclitaxel and tetrandrine for overcoming multidrug resistance. Int J Pharm 489, 318–330. 25956050 10.1016/j.ijpharm.2015.05.010

[47] Tang H , Zeng L , Wang J , Zhang X , Ruan Q , Cui S and Yang D (2017) Reversal of 5‐fluorouracil resistance by EGCG is mediate by inactivation of TFAP2A/VEGF signaling pathway and down‐regulation of MDR‐1 and P‐gp expression in gastric cancer. Oncotarget 8, 82842–82853. 29137307 10.18632/oncotarget.20666 PMC5669933

[48] Farabegoli F , Papi A , Bartolini G , Ostan R and Orlandi M (2010) (‐)‐Epigallocatechin‐3‐gallate downregulates Pg‐P and BCRP in a tamoxifen resistant MCF‐7 cell line. Phytomedicine 17, 356–362. 20149610 10.1016/j.phymed.2010.01.001

[49] Gu JW , Makey KL , Tucker KB , Chinchar E , Mao X , Pei I , Thomas EY and Miele L (2013) EGCG, a major green tea catechin suppresses breast tumor angiogenesis and growth via inhibiting the activation of HIF‐1alpha and NFkappaB, and VEGF expression. Vasc Cell 5, 9. 23638734 10.1186/2045-824X-5-9 PMC3649947

[50] Wang W , Xiong X , Li X , Zhang Q , Yang W and Du L (2019) In silico investigation of the anti‐tumor mechanisms of epigallocatechin‐3‐gallate. Molecules 24, 1445. 10.3390/molecules24071445 PMC6480119 30979098

[51] Shi J , Liu F , Zhang W , Liu X , Lin B and Tang X (2015) Epigallocatechin‐3‐gallate inhibits nicotine‐induced migration and invasion by the suppression of angiogenesis and epithelial‐mesenchymal transition in non‐small cell lung cancer cells. Oncol Rep 33, 2972–2980. 25845434 10.3892/or.2015.3889

[52] Seeliger H , Guba M , Kleespies A , Jauch KW and Bruns CJ (2007) Role of mTOR in solid tumor systems: a therapeutical target against primary tumor growth, metastases, and angiogenesis. Cancer Metastasis Rev 26, 611–621. 17713840 10.1007/s10555-007-9077-8

[53] Horsman MR and Vaupel P (2016) Pathophysiological basis for the formation of the tumor microenvironment. Front Oncol 6, 66. 27148472 10.3389/fonc.2016.00066 PMC4828447

